# A New Gene Therapy Approach for Cardiovascular Disease by Non-coding RNAs Acting in the Nucleus

**DOI:** 10.1038/mtna.2014.48

**Published:** 2014-11-18

**Authors:** Tiia Husso, Seppo Ylä-Herttuala, Mikko P Turunen

**Affiliations:** 1 Department of Biotechnology and Molecular Medicine, A.I.Virtanen Institute, University of Eastern Finland, Kuopio, Finland; 2 Science Service Center and Gene Therapy Unit, Kuopio University Hospital, Kuopio, Finland; 3 Department of Molecular and Experimental Medicine, The Scripps Research Institute, La Jolla, California, USA

**Keywords:** cardiovascular, epigenetics, non-coding RNA, transcriptional gene activation, transcriptional gene silencing

## Abstract

This review discusses recent developments in the use of non-coding RNAs (ncRNAs) for the regulation of therapeutically relevant genes, with special focus on applications for the treatment of cardiovascular diseases. The interest in using ncRNAs as therapeutics has steadily increased since the discovery of RNA interference. During the last decade it has become evident that these RNAs, delivered either as oligos or expressed as small hairpin RNAs (shRNAs) from vectors, can either upregulate (transcriptional gene activation, TGA) or downregulate (transcriptional gene silencing, TGS) gene expression, typically inducing epigenetic changes in their target sites in the chromatin. Also, the important role of naturally occurring long non-coding RNAs (lncRNAs) has been recently discovered and will likely provide new insights into cardiovascular pathology and provide new treatment strategies based on the manipulation of their expression. In this review, we discuss the possibility of using ncRNAs for activating or silencing therapeutically relevant genes, such as *VEGF-A*, for the treatment of cardiovascular disease.

## Introduction

According to the reports by WHO, cardiovascular diseases (CVDs) are responsible for the majority of the deaths worldwide and their prevalence is increasing (http://www.who.int/mediacentre/factsheets/fs317/en/). Traditional pharmacological treatments for CVD have limited efficiency and an unmet clinical need exists for novel treatment strategies, such as gene therapy. The recently published ENCODE project^1^ has revealed the existence of vast amount of non-coding RNAs (ncRNAs) in human cells, and the role of ncRNAs in both basic cellular processes and diseases has in recent years gained much interest. Understanding the complexity of various RNA species, their interactions and intra- and extracellular trafficking will provide a better understanding of the biological basis of various diseases. Furthermore, manipulation of these ncRNAs represents a promising new gene therapy strategy for the treatment of various diseases, including CVD.^2^

## Mechanistic Aspects of Gene Regulation by ncRNAs

**Figure 1:**
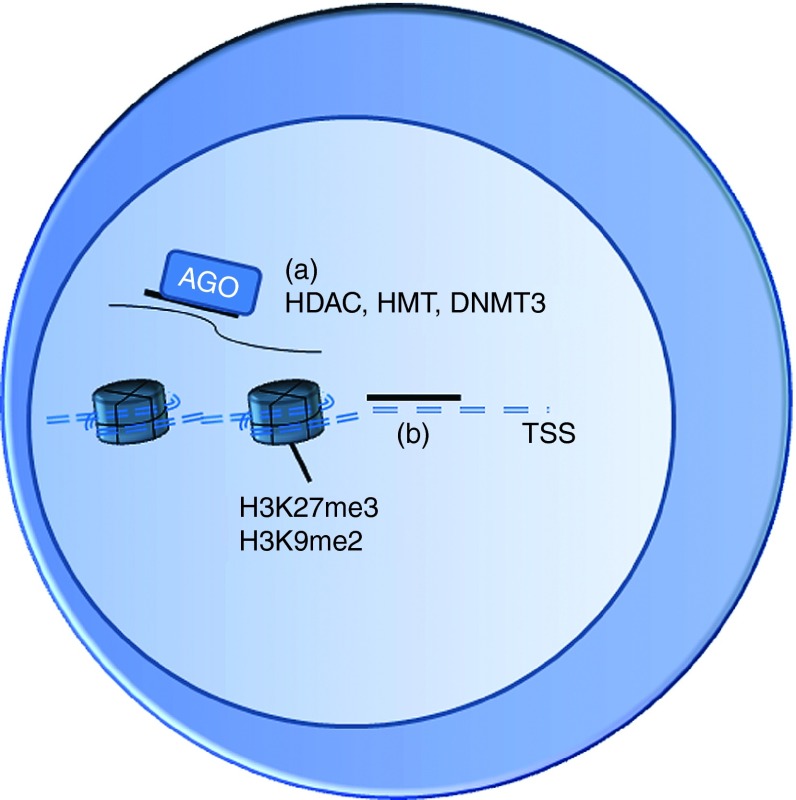
**Possible mechanisms of transcriptional gene silencing (TGS).** (**a**) Small RNA is targeted to a promoter-associated transcript. Epigenetically active enzymes (histone deacetylases, histone methyltransferases and DNA methyltransferase 3) are recruited and their activity leads to histone modifications such as histone 3 lysine 9 dimethylation (H3K9me2) and lysine 27 trimethylation (H3K27me3). These histone modifications lead to the silencing of transcription and heterochromatin formation. (**b**) The small RNA can target the transcriptional start site (TSS) as such and prevent transcription by forming DNA:RNA triplexes.

**Figure 2:**
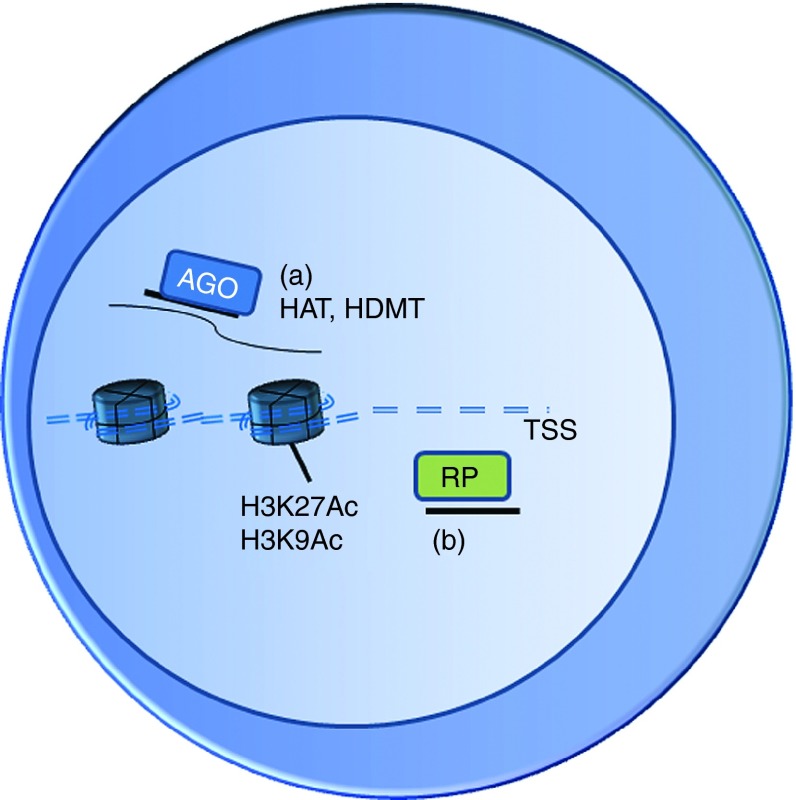
**Possible mechanisms of transcriptional gene activation (TGA).** (**a**) Argonaute (AGO)-bound small RNA binds to promoter-associated transcripts and recruites epigenetically active enzymes, such as histone acetylases and histone demethylases. Their activity leads to histone modifications such as histone 3 lysine 9 acetylation (H3K9Ac) and lysine 27 acetylation (H3K27Ac), which are markers for active transcription. (**b**) Small RNA could also inhibit the activity of repressor protein and allow transcriptional activity.

Many of the small ncRNAs have been considered to function at the post-transcriptional level in the cytoplasm, mediating, for example, mRNA degradation and inhibition of mRNA translation. The RISC protein complex, guided by the small RNA (eg miRNA or siRNA) to the target mRNA sequence, includes the effector proteins such as Argonaute protein family member AGO2 that possesses endonuclease activity. Recently many of these components have been observed also in the nucleus^3^ and small RNAs complementary to gene promoter sequences have been shown to regulate gene expression. Besides TGS, promoter-targeted small RNAs are also able to activate gene expression. Small RNA-mediated gene regulation involves epigenetic changes on the promoter and the effect is potentially long-lasting (**Figures 1** and **2**).^4^ Various reports have related increased levels of active chromatin markers, such as H3K4me2/me3, and increased RNA polymerase II occupancy to TGA and similarly increased levels of repressive chromatin markers, such as HK9me2 and lysine 27 trimethylation (H3K27me3), and decreased polymerase occupancy to TGS.^5,6,7,8,9^ Involvement of other proteins has also been shown in these processes, for example, the Argonaute proteins (both AGO1 and AGO2) have been shown in many reports of TGS^4,7,10^ and TGA,^11,12,13,14^ and in TGA the recruitment of HP1γ (a paralog of Heterochromatin Protein 1) to the promoter is decreased.^15^

The exact mechanisms for TGS and TGA are still not clear but a few different mechanisms have been suggested; small RNAs targeted to the promoter sequences may be in direct contact with the chromatin forming DNA–RNA hybrids^16^ or they might bind to non-coding sense or antisense transcripts located at the target site forming RNA-RNA hybrids.^15,17^ It is also possible that the antisense transcripts could serve as a scaffold where the small RNA guided complex recruit chromatin-modifying proteins.^18^ It has been shown that the same gene can be either activated or suppressed by a small RNA in a sequence-dependent manner.^5^ These effects are also cell type specific and may depend on the basal expression level of the targeted gene and the presence of the antisense transcripts on the promoter.^5,12,15,19^ For example, MS1 and C166 are both mouse endothelial cells, but typically only C166 cell line is responsive to TGS/TGA by promoter-targeted small hairpin RNAs (shRNAs). However, if these cells are pretreated with 5-azacytidine, a DNA methyltransferase inhibitor, MS1 cells become responsive to TGS, whereas C166 cells turn unresponsive.^20^ Therefore, the preexisting epigenetic state of the cell is important for the function of nuclear small RNA-mediated gene regulation.^9,19,20^

Small RNAs have also been shown to affect alternative splicing events, in a mechanism similar to TGS. siRNAs targeted to sequences near an alternative exon regulated the splicing of that exon.^21^ This involved an increase in histone 3 lysine 9 dimethylation (H3K9me2) and H3K27me3 levels at the target site within the gene, AGO1 and HP1α recruitment and a change in the RNA polymerase II elongation rate.^21^ This finding was supported by another study where it was shown that the nuclear AGO1 and AGO2 directly associate with both chromatin-modifying proteins and regulators of alternative splicing, and these chromatin-associated complexes increase H3K9me3 amount and HP1γ binding, thus slowing down the RNA polymerase II elongation rate and facilitating inclusion of variant exons.^22^ It is interesting to note that in the study by Alló *et al*., the presence of the antisense transcripts on the siRNA target site also affected the activity of siRNA and contributed to the differences between cell lines.^21^

Most of the reports have utilized synthetic small double-stranded RNAs, but these studies imply that there may also be a similar endogenous gene regulation mechanism. Some bioinformatic studies have already predicted that miRNAs have target sites on gene promoters in similar quantity as in the 3'UTRs and with very high complementary.^23,24^ There are also some experimental data supporting these computational analyses. For example, Place *et al*. identified a target site for miR-373 on *E-cadherin* promoter and both miR-373 mimic and synthetic oligo fully complementary to the same sequence were able to induce *E-cadherin* expression.^6^ Younger and Corey screened the *progesterone receptor* (PR) promoter antisense transcript for potential miRNA binding sites and found four different miRNAs that were able to inhibit PR expression when transfected to cells.^25^ These experiments demonstrated that while synthetic siRNAs inducing either TGS or TGA need to be fully complementary to their target promoter, the endogenous miRNAs can include even extensive mismatches and still achieve to regulate the gene transcription, making the prediction of functional target sites of miRNAs especially difficult. A study by Dharap *et al*.^26^ found that miR-324-3p is induced 6 hours after focal cerebral ischemia, and the *in silico* study predicted a strong binding site for this miRNA on the *RelA* promoter. RelA is a subunit of NF-κB and is activated after cerebral ischemia.^27^ The *in vitro* studies showed that the expression of *RelA* was indeed induced with transfection of pre-miR-324-3p to cells of neural origin, indicating that miR-324-3p may be regulating *RelA* expression in ischemia in TGA-like manner.^28^ It has also recently been shown that miRNAs regulate the function and expression of both other miRNAs and their own and for example, in *Caenorhabditis elegans*, it was also shown that miRNA *lin-4* is able to autoregulate its own expression by increasing the transcription through a complementary target site on its promoter.^29^

Additionally, lncRNAs have been reported to regulate gene transcription and promoter epigenetics. A study by Khalil *et al*.^30^ showed that lncRNAs are bound by PCR2, a protein complex responsible for trimethylation of H3K27, and other chromatin-modifying complexes such as CoREST, suggesting that lncRNAs regulate gene transcription by directing these proteins to specific sites in the genome. In mouse embryonic stem cells, lncRNAs Evx1as and Hoxb5/6as were shown to be associated with MLL1, a histone methyltransferase that trimethylates H3K4, suggesting that they are involved in epigenetically directing differentiation.^31^ ANRIL, a lncRNA produced from a genomic locus considered a hotspot for disease-associated polymorphisms that have been often associated with, *e.g*., CVD and diabetes, has been shown to associate with both CBX7 (a member of PRC1 complex) and SUZ12 (a PRC2 member).^32,33^ The overexpression of ANRIL also downregulates the expression of a set of chromatin-modifying proteins such as histone acetyltransferase p300.^34^ In mouse embryonic stem cells, an ANRIL variant p15AS (p15 antisense transcript) induced p15 silencing through heterochromatin formation and DNA methylation after differentiation.^35^ Experiments in human aortic vascular smooth muscle cells showed that ANRIL may have different splice variants that guide distinct gene regulation patterns, modulating the expression of several genes related to the pathogenesis of atherosclerosis.^36^ LncRNA Fendrr regulates heart development in embryos by epigenetic mechanisms.^37^ This involves the action of histone-modifying complexes PRC2 and TrxG/MLL that regulate the activation of genes that are pivotal to developmental functions. Specifically, Fendrr anchors PRC2 at its target promoters and hence increases PRC2 occupancy and H3K27 trimethylation leading to the silencing of target gene expression.^37^ In a recent study of mouse cardiac long non-coding transcriptome after myocardial infarction, many novel lncRNAs were identified and the differential expression was correlated with physiology.^38^ These heart-specific novel lncRNAs were often associated with active enhancers, which act as cell type-specific regulatory elements, and also with miRNA networks previously shown to influence cardiac stress response, indicating a complex regulatory system of different ncRNAs.

## Therapeutic Possibilities by Using ncRNAs: Focus on Cardiovascular Disease

**Figure 3:**
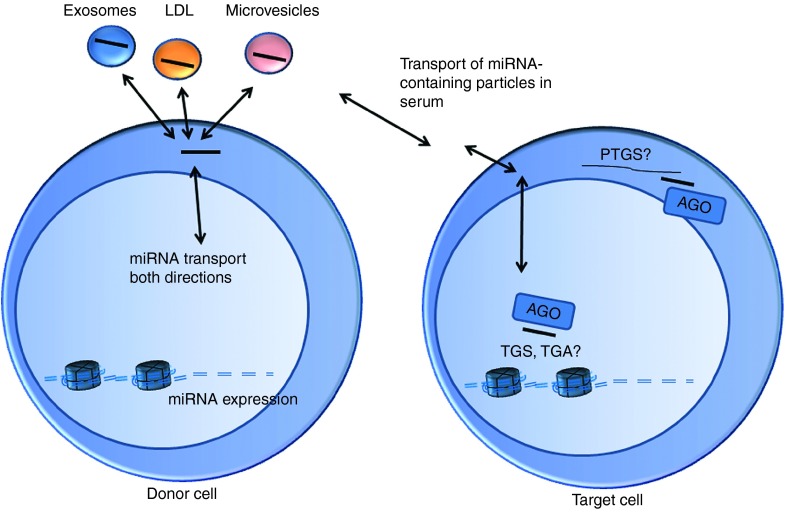
**Small RNAs, such as miRNAs, have both cytoplasmic (PTGS) and nuclear functions.** They can be secreted to extracellular space in microvesicles, low-density lipoprotein (LDL) particles or exosomes and can be internalized by recipient cells. Their functions in recipient cells are still unclear, but can include PTGS or TGS/TGA.

A common pathological feature of CVD is the harmful proliferation of smooth muscle cells. It has been shown that the proliferation of smooth muscle cells is at least partly an epigenetic process and that this proliferation can be inhibited by preventing histone deacetylase activity using siRNAs.^39^ In addition to these classical RNAi treatment strategies, new therapy concepts based on nuclear action of ncRNAs have evolved during the past years. We have recently shown that the upregulation of *VEGF-A* gene using a promoter-targeted shRNA significantly decreases the infarct size in mouse myocardial infarction model^20^ and improves blood flow in ischemic hindlimb through increased vasculature.^5^ The observed therapeutic efficiencies in both studies were surprisingly good, considering the limited *in vivo* transduction efficiency of lentiviral vectors. There are several possible reasons for these observed effects. First, VEGF-A is a secreted protein and can exert its effects from transduced cells to the surrounding tissue.^40^ Second, all isoforms of VEGF-A are upregulated in response to the epigenetic regulation of endogenous gene^20^ and this will likely provide more natural responses in the target tissue as compared with traditional gene therapy approach where usually only one isoform of the therapeutic gene is delivered, typically driven by a strong promoter. The endogenous gene encoding human VEGF-A consists of eight exons and alternative splicing produces at least four different isoforms (VEGF-121, VEGF-165, VEGF-189, and VEGF-206). These different isoforms differ in their biological functions, such as in heparin binding that in turn determines how strongly the isoform binds to extracellular matrix and can therefore be secreted to neighboring cells.^41^ The third and most intriguing possibility (**Figure 3**) is that the expressed shRNAs are secreted out of the cells and taken up by neighboring recipient cells, where they pass on the regulatory effects. These shRNAs structurally resemble miRNAs that have been shown to facilitate communication between endothelial cells and smooth muscle cells via an extracellular-vesicle-mediated mechanism in response to atheroprotective stimuli.^41^ Furthermore, Vickers *et al*.^42^ showed that miRNAs are transported in plasma by high-density lipoprotein and taken up by the recipient cells. They found several interesting miRNAs of which miR-223 was one of the most abundant and it's expression was increased in atherosclerosis. This miRNA is highly conserved^43^ and is involved in the regulation of many biological processes.^44,45^ miRNAs can also be secreted in apoptotic bodies by endothelial cells during atherosclerosis, which in turn induces the recruitment of progenitor cells in atherosclerotic mice.^46^ The secretion of miRNAs in exosomes has been shown and for example endothelial cells can transfer miRNAs to cardiomyocytes in some pathological conditions.^47^ It is tempting to think that therapeutic ncRNAs could be delivered *in vivo* using similar, even synthetic, vehicles as carriers. It has already been shown that exosomes produced *in vitro* can be delivered *in vivo* in an acute mouse myocardial ischemia/reperfusion model.^48^

## Conclusions and Future Prospects

Epigenetic gene regulation allows organisms, including humans, to adapt to the environmental stress and changing living conditions.^2^ However, the emergence of CVDs on global scale is constantly growing and these pathologies can be partially related to epigenetic regulation of genes and regulatory RNAs, such as miRNAs or other ncRNAs. Therefore, it is an intriguing approach to target these mechanisms by gene therapy strategies. One such method for regulating the expression of a specific gene is to use small RNAs that are complementary to gene promoters, delivered either as RNA oligonucleotides or as hairpin RNAs using vectors. Even though the gene therapy field still has many obstacles to be solved, such as limited efficiency of transfection/transduction and immune responses against vectors and/or transgenes,^49^ the manipulation of naturally occurring, ncRNA-mediated, regulatory mechanisms may provide more efficient biological responses and allow using lower vector amounts *in vivo*. When using the traditional gene delivery as a treatment strategy, there often exists a need to regulate the expression of the transgene to avoid unwanted responses, such as cancer when using potent growth factors. Short-term delivery of small RNAs that mediate their effects by epigenetic mechanisms may also help to avoid these problems, since the therapy will merely give a push in the cell towards the desired response and the epigenetic status of the cell will later better accommodate to the long-term status and needs of the tissue. Thus, characterizing the nature and functions of ncRNAs will increase the overall understanding of the mechanisms involved in CVDs and help to develop novel treatment strategies based on gene therapy.
